# Cephalic Index Variation in the Indigenous Population of Tribal Districts in Himachal Pradesh

**DOI:** 10.7759/cureus.68018

**Published:** 2024-08-28

**Authors:** Nirmal Nagar, Yatiraj Singi, Dipen Dabhi, Reshma Rathore

**Affiliations:** 1 Department of Forensic Medicine and Toxicology, India Institute of Medical Sciences Bilaspur, Bilaspur, IND

**Keywords:** mesocephalic, brachycephalic, high altitude, indigenous population, tribal population, head shapes, races, craniometry, anthropology, cephalic index

## Abstract

Introduction

The cephalic index (CI) is the most commonly used index to determine the race of an individual. It is calculated as the ratio of the breadth of the skull to the length of the skull multiplied by 100. The CI of an individual can be influenced by factors such as race, ethnicity, genetic predisposition, lifestyle choices, nutritional habits, environmental factors, and climatic conditions.

Material and methods

This study was carried out on 413 individuals in the tribal districts of Himachal Pradesh (Kinnaur, Spiti, and Lahaul), with 247 (59.8%) male and 166 (40.2%) female subjects. Measurements of the skull were taken using a spreading caliper. The CI was calculated using Hardlika’s method and data were analyzed using IBM SPSS version 27 (IBM Corp., Armonk, NY, US).

Results

The head shapes of the majority of the tribal population of Himachal Pradesh are mesocephalic, with the mean CI for males and females being 78.90 and 79.81, respectively, without any significant difference. The mean CI of the overall population is 79.27. The majority population of the Kinnaur district is brachycephalic, while the Spiti and Lahaul populations are mesocephalic with females predominately brachycephalic and males predominately mesocephalic.

Conclusion

The Indigenous population of Himachal Pradesh is mesocephalic with females predominately brachycephalic.

## Introduction

Cephalometry is the analysis of anthropometric measurements of the human head. This holds considerable significance in several areas such as identifying unknown living individuals, determining the identity of individuals from skull specimens, discerning racial characteristics, employing 3D reconstruction imaging for identification purposes, and aiding in the resolution of criminal cases [1].

The cephalic index (CI), also known as the cranial index or breadth index, is widely recognized as a primary metric for human racial classification. Anders Retzius, a Swedish Anatomy professor, first introduced the CI, which was initially employed to identify human remains found in Europe [2].

The importance of age, gender, and population-specific CI data extends across various domains, offering valuable insights for monitoring treatment and predicting orthodontic outcomes. Moreover, this knowledge holds significance in plastic and reconstructive surgeries, particularly those addressing craniofacial deformities [3]. In contemporary contexts, the CI is also utilized to define the physical attributes of individuals and to estimate the age of fetuses for legal and obstetrical considerations [4,5]. Furthermore, it serves as a foundation for diagnostic prediction, such as in cases of dolichocephalic individuals, who are less susceptible to otitis media, and in individuals with Apert's syndrome, who exhibit hyperbrachycephalic characteristics [6,7].

This study presents a CI database crucial for forensic medicine, plastic surgery, and orofacial reconstruction. It is the first such study to explore the CI in the adult Indigenous population of Kinnaur, Lahaul, and Spiti districts, Himachal Pradesh, considering age, gender, and geography.

## Materials and methods

This study was approved by the Institute Ethics Committee (Biomedical & Health Research) at AIIMS Bilaspur, reference no. 68/23, dated 05/08/2023. This prospective cross-sectional study was conducted among the adult population of the tribal districts of Kinnaur, Lahaul, and Spiti in Himachal Pradesh, India. Participants were selected from families that have resided in these tribal districts for at least three generations, ensuring that our study population represents the inherited characteristics of the native tribal population.

According to the last Census in 2011, the total population of these tribal districts is 115,865. A sample size of 383 was calculated to achieve a 95% confidence level with a 5% margin of error. All participants were aged between 18 and 50 years and selected through cluster sampling via a door-to-door survey. Participants with visible deformities in the head, face, or vertebrae were excluded, as were those with a history of craniofacial injury, surgery, or developmental or metabolic disorders. To minimize inter-observer bias, all measurements were taken by a single observer using standard anthropometric instruments, following the technique described by Vallois [8].

**Figure 1:**
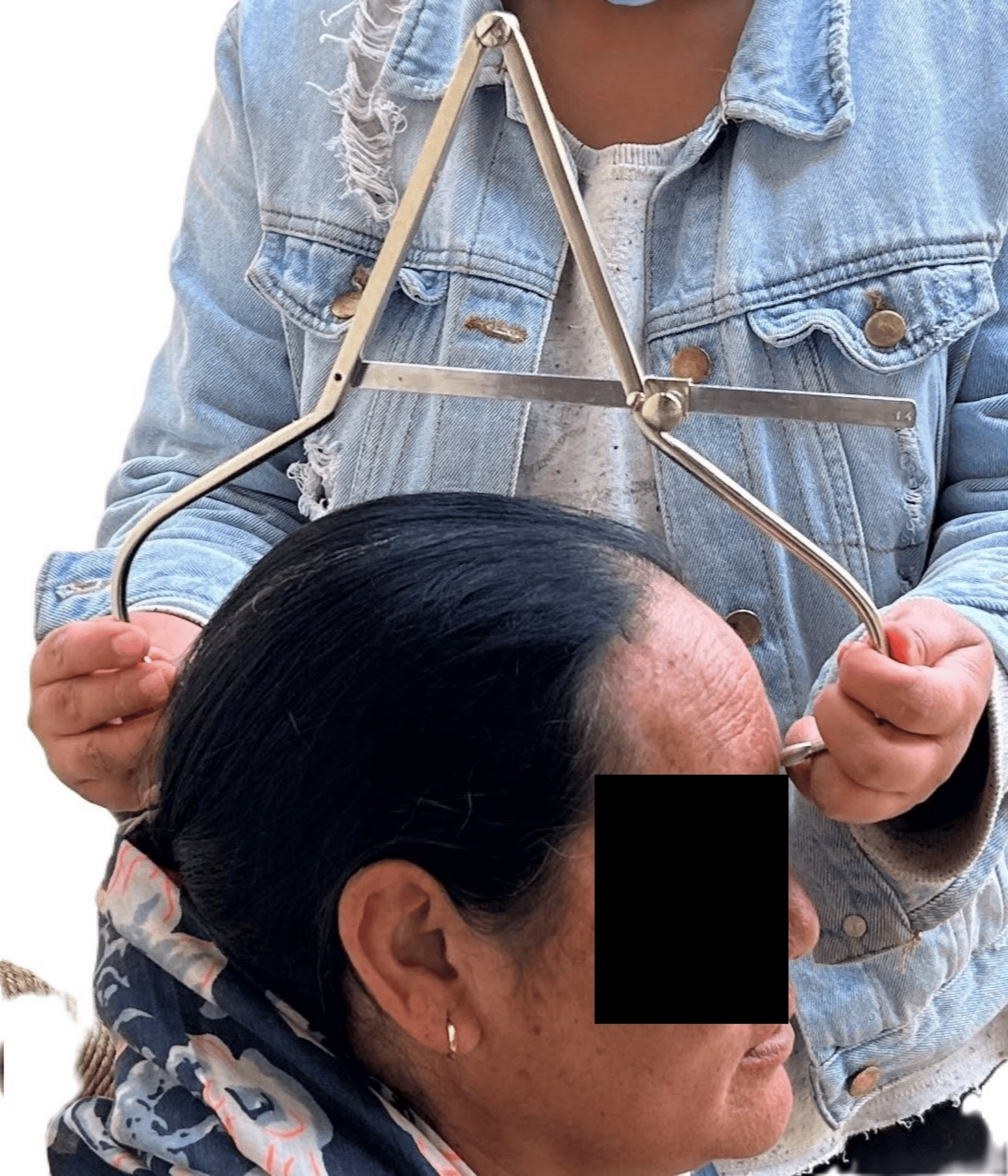
Measuring head length

All measurements were taken using a spreading caliper, thrice in a single sitting on the same day to avoid observational bias, and the average of the three recordings was used for analysis. Subjects were sitting in a relaxed position, with their heads anatomically aligned in the orbito-ocular plane (horizontal Frankfurt plane). The glabella (G) is the point above the nasal root between the eyebrows intersected by the mid-sagittal plane; the opisthocranion (OP) is the most posterior point on occipital protuberance of the head in the mid-sagittal plane; and the Euryon (Eu) is the most lateral point on the sides of the head, indicating maximum cranial breadth. Head length is determined as the maximal anteroposterior diameter from the glabella to the opisthocranion (Figure 1).

**Figure 2:**
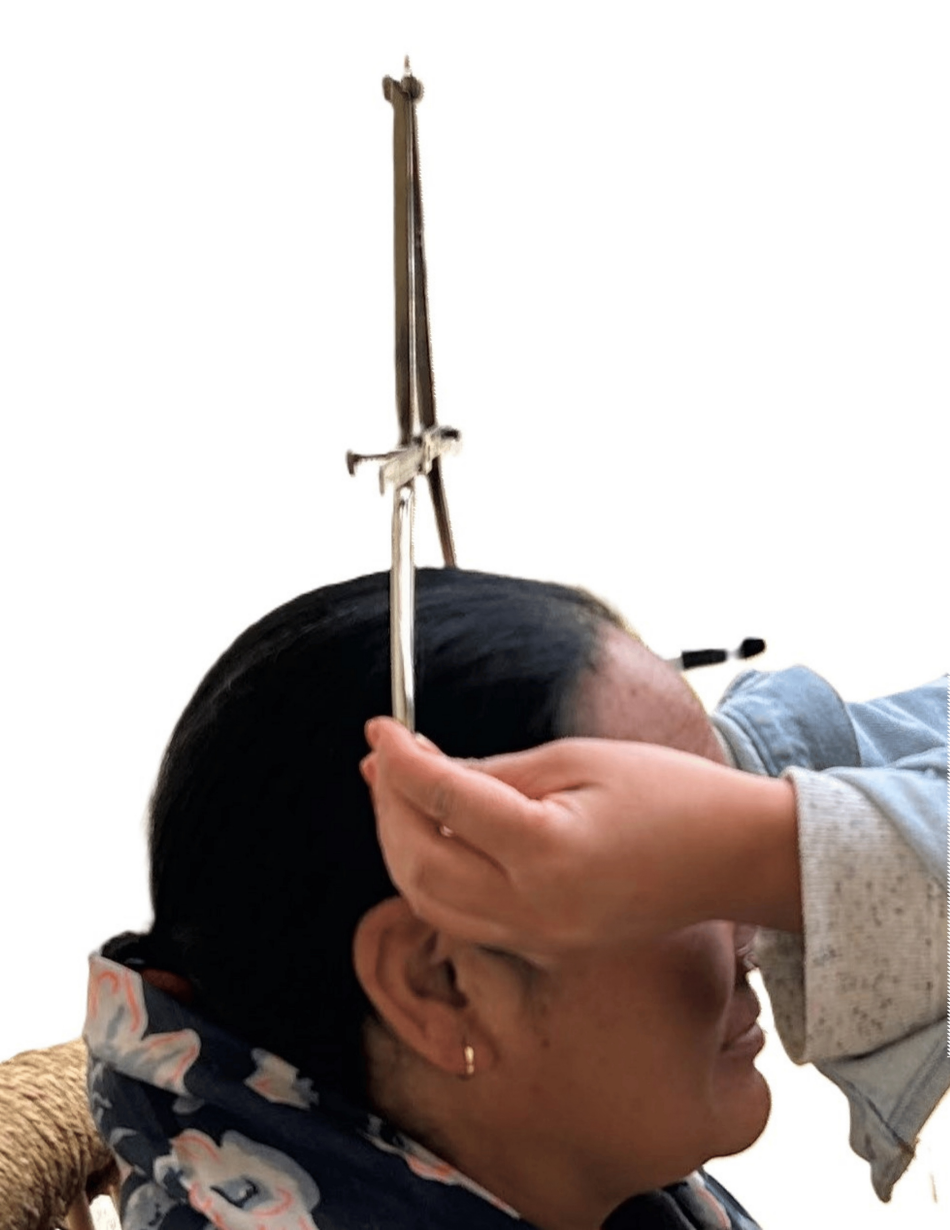
Measuring head breadth

The head breadth is measured as the maximum transverse diameter between the two Euryons (Figure 2).

The CI was assessed using the method described by Hardlika, as this method has been used numerous times by researchers [9]. According to it, the cephalic index (CI) was calculated using the formula: CI = [Cranial breadth / Cranial length] × 100. Based on the CI, head shapes are classified into three main types: dolichocephalic or long-headedness (CI < 75); brachycephalic or short and broad-headedness (CI > 80); and mesocephalic (CI = 75-80) [10].

Data entry was completed in Microsoft Excel (Microsoft Corp., Redmond, WA, US) while data analysis was conducted using IBM SPSS version 27 (IBM Corp., Armonk, NY, US). Descriptive statistics, such as mean, standard deviation, median, and minimum and maximum values, were calculated. For parametric data, an independent samples t-test was used for comparisons between two groups, and one-way analysis of variance (ANOVA) was applied for comparisons among more than two groups. Categorical data were analyzed using the chi-squared test. P value less than 0.05 is considered to be significant.

## Results

CI was calculated for both genders and across the different districts within the studied population. Interestingly, the mean CI for males, females, and the overall population falls within the mesocephalic category. Notably, the mean CI for females is slightly higher than that for males. However, it's intriguing that despite this discrepancy, there are no statistically significant gender differences present (P value= 0.079) (Table 1).

**Table 1:** The variation in CI among the tribal population of Himachal Pradesh CI: cephalic index

Parameters	Male	Female	Total
Number (%)	247(59.8%)	166(40.2%)	413(100%)
Mean + SD	78.90 + 5.27	79.81 + 4.91	79.27 + 5.13
Median	78.14	79.48	79.03
Minimum	66.84	68.9	66.84
Maximum	112.88	106.94	112.88

**Figure 3:**
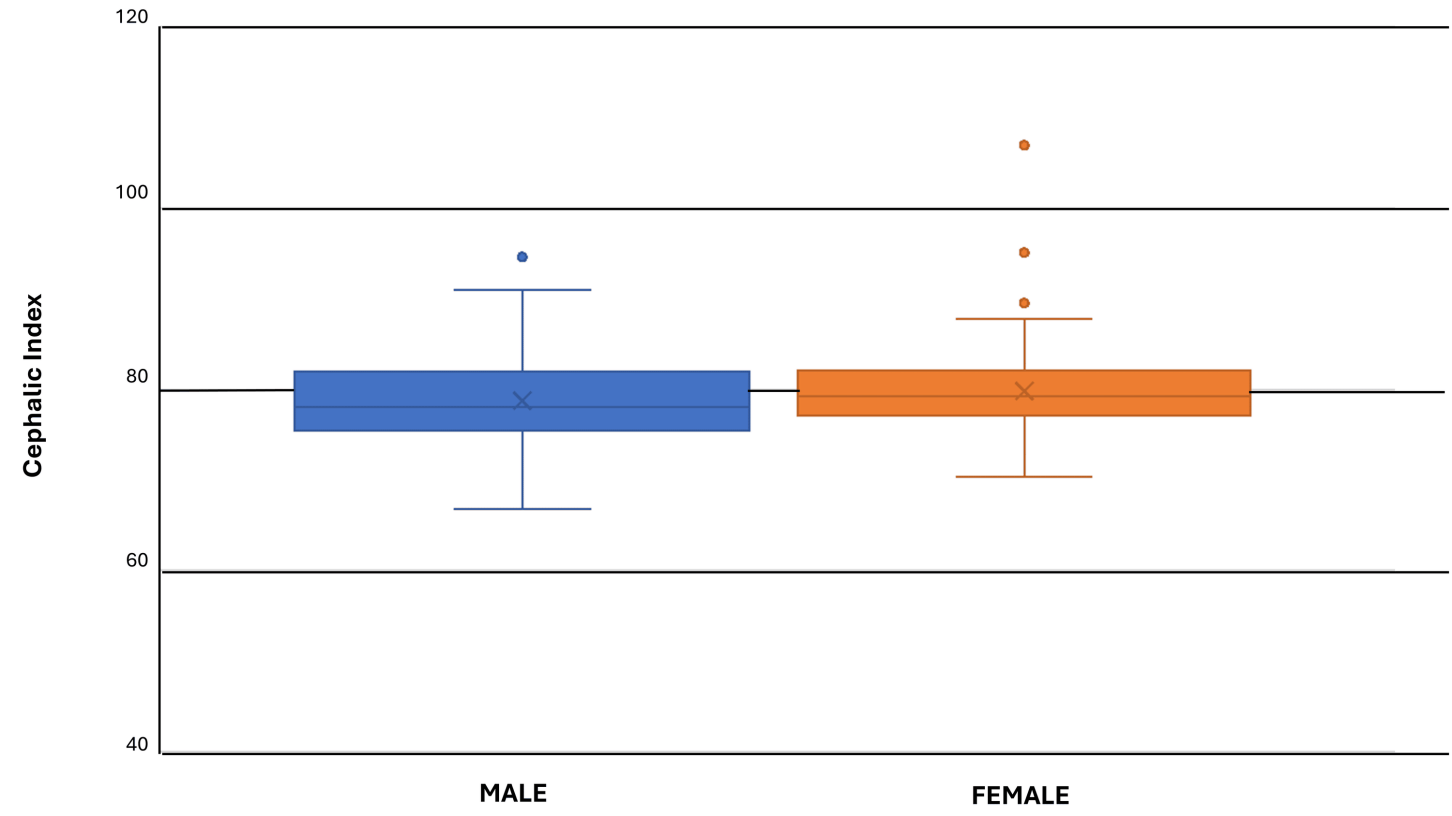
CI variation in both genders CI: cephalic index

A box and whisker plot was created to illustrate the variation in CI for both males and females (Figure 3). Ninety-nine percent of the study population is within normal limits and less than 1% of the study subjects were outliers, which did not affect the final result.

In Kinnaur, males exhibit a mean CI categorized as mesocephalic while females show a tendency toward brachycephaly. Conversely, both males and females in Spiti and Lahaul have a mean CI falling within the mesocephalic range. Overall, the mean CI across all districts tends toward mesocephalic but is gradually approaching brachycephalic. Notably, there is no significant difference observed in the mean CI between Kinnaur and Spiti (P value 0.775), Kinnaur and Lahaul (P value 0.208), and Spiti and Lahaul (P value 1.00) (Table 2).

**Table 2:** District-wise variation in CI CI: cephalic index

Parameters	Kinnaur			Spiti			Lahaul		
	Male	Female	Total	Male	Female	Total	Male	Female	Total
No. (%)	125	102	227 (55%)	53	46	99 (24%)	69	18	87 (21%)
Mean + SD	79.38 + 4.61	80.05 + 4.97	79.68 + 4.79	78.47 + 5.09	79.57 + 3.8	78.98 + 4.57	78.37 + 6.3	79.03 + 6.54	78.5+ 6.36
Median	79.1	79.72	79.46	77.72	79	78.46	77	79.23	77.3
Minimum	67.52	68.89	67.52	66.84	72.1	66.84	68.96	70.62	68.96
Maximum	94.61	106.94	106.94	100	87.86	100	112.88	95.1	112.88

In Kinnaur, the predominant population exhibits brachycephalic traits, whereas in Spiti and Lahaul, the majority are categorized as mesocephalic. However, when considering the overall population, males tend to be predominantly mesocephalic while females lean toward brachycephaly. When both sexes are combined, the overall population shows a tendency toward mesocephalic traits (Table 3).

**Table 3:** Percentage-wise variation of CI among districts CI: cephalic index

Districts	Parameters	Dolichocephalic (<75)	Mesocephalic (75- 79.99)	Brachycephalic (>80)
Kinnaur	Male (n, %)	20 (16%)	51 (40.8%)	54 (43.2%)
	Female (n, %)	13 (12.75%)	40 (39.22%)	49 (48.04%)
	Total (n, %)	33 (14.54%)	91 (40.09%)	103 (45.37%)
Spiti	Male (n, %)	11 (20.75%)	26 (49.06%)	16 (30.19%)
	Female (n, %)	4 (8.7%)	23 (50%)	19 (41.3%)
	Total (n, %)	15(15.15%)	49 (49.49%)	35 (35.35%)
Lahaul	Male (n, %)	20 (28.99%)	28 (40.58%)	21 (30.43%)
	Female (n, %)	5 (27.78%)	7 (38.89%)	6 (33.33%)
	Total (n, %)	25 (28.74%)	35 (40.23%)	27 (31.03%)
Overall	Male (n, %)	51 (20.65%)	105 (42.51%)	91 (36.84%)
	Female (n, %)	22 (13.25%)	70 (42.17%)	74 (44.58%)
	Total (n, %)	73 (17.67%)	175 (42.37%)	165 (39.95%)

## Discussion

The CI of an individual can be influenced by factors such as race, ethnicity, genetic predisposition, lifestyle choices, nutritional habits, environmental factors, and climatic conditions [11]. Australian Aborigines and native Southern Africans typically exhibit dolichocephalic characteristics while Europeans and Asians tend to have mesocephalic features. On the other hand, Mongolians and Andaman Islanders often display brachycephalic traits [3]. The resemblance in the cephalic index among parents, offspring, and siblings can provide insights into the hereditary pattern of the cephalic index [2].

We compared the cephalic index (CI) findings from our study with data from other regions of India to explore correlations and variations in CI values across different areas (Table 4).

**Table 4:** A comparison of CI in the present study with populations of different regions of India CI: cephalic index

Authors	Year	People / Country	CI (M)	CI(F)	CI (Mean)
Shah GV et al. [12]	2003	Gujarat	80.42	81.2	80.81
Mahajan A et al. [13]	2009	Punjab	81.34	85.75	83.55
Salve VM et al. [14]	2011	Andhra Pradesh	75.68	78.2	76.94
Khair S et al. [15]	2011	Mumbai region of Maharastra	81.28	75.22	78.48
Jhadav et al. [16]	2011	Different casts in Gujrat	80.20	--	--
Yagain et al. [17]	2012	Indian students	77.85	80.85	79.35
Gupta et al. [18]	2013	North India	78.79	76.83	77.81
Uttekar et al. [19]	2013	South Gujrat	82.82	82.48	82.65
Kumar M et al. [20]	2013	Bania caste in Haryana	66.72	72.25	69.49
Nair et al. [2]	2014	Madhya Pradesh	81.03	80.31	81.21
Mishra M et al. [21]	2014	Vindhya region of Madhya Pradesh	79.85	79.05	79.45
Shah T et al. [22]	2015	Gujrat	77.15	77.38	77.26
Jaiswal P et al. [23]	2016	Hadoti region of Rajasthan	77.60	78.47	78.04
Sultan SI et al. [24]	2017	Marathwada region of Maharastra	79.12	78.67	78.89
Gosh SM et al. [25]	2018	West Bengal	81.2	80.76	81.09
Ahmed et al. [26]	2018	Jhalawar region of Rajasthan	74.07	74.39	74.23
Byhnadaorili et al. [27]	2018	Tribes of Northeast states	83.08	82.90	82.99
Nawaz Ahmed SK et al. [28]	2019	Kelambakkam region of Tamil Nadu	77.93	75.22	76.58
Khanduri et al. [29]	2021	North India	75.59	77.94	76.67
Present study	2023	Tribal districts of Himachal Pradesh	78.90	79.81	79.27

The mean CI for the Indian population, including data from our study, falls within the mesocephalic range [14,15,20-24,26,29]. Our analysis reveals that the mean CI for females is consistently higher than that for males, which corresponds with the majority of studies reviewed [12-14,17-23,26-27,29]. Furthermore, our study shows that most females exhibit a brachycephalic pattern, a finding that is corroborated by other research in the field [2,12-13,17,19,25,27].

Kinnaur and its neighboring district share a border with the Tibet region, with multiple routes connecting these regions. These routes have historically facilitated the exchange of culture and religion, as well as the migration of people. This migration led to the intermingling of the Mongolian race with the predominantly mesocephalic Indian race [30]. This can explain the predominant brachycephalic CI in the Kinnaur district and adjoining areas.

Limitations of the study

This study specifically targeted the tribal population of Himachal Pradesh. Therefore, it's important to note that the CI calculated from this study may not be generalizable to the entire population of Himachal Pradesh. The CI of an individual is influenced by a multitude of factors, including age, environmental conditions, nutrition, and lifestyle, among others. While these variables were not the primary focus of our study, it's important to note that our research centered on a population residing in the region for the past three generations. Exploring these parameters in greater detail could offer valuable insights in future investigations.

## Conclusions

The head shape of the tribal population of Himachal Pradesh is predominantly mesocephalic, with a mean CI of 78.90 for males and 79.81 for females, resulting in an overall population mean CI of 79.27. While the majority of males exhibit mesocephalic traits, females tend toward brachycephalic characteristics. However, when considering the overall population, mesocephalic traits are predominant. Specifically, the population of Kinnaur tends to be predominantly brachycephalic while people from Spiti and Lahaul exhibit mesocephalic tendencies.
